# Analgesic Effect of Ultrasound-Guided Caudal Block Versus Quadratus Lumborum Plane Block in Lumbar Spine Surgery in Adult Patients: A Double-Blinded Prospective Comparative Study

**DOI:** 10.5812/aapm-169635

**Published:** 2026-02-25

**Authors:** Alyaa Abdel Sattar Mohamed Hassan, Atef Mohamed Mahmoud, Samar Ahmed Ramadan Mohamed, Mohammed Awad Alsaied, Ahmed Ali Lotfy

**Affiliations:** 1 Faculty of Medicine, Fayoum University, Fayoum, Egypt

**Keywords:** Anesthesia, Caudal, Nerve Block, Quadratus Lumborum, Ultrasonography, Postoperative Pain, Analgesics, Opioid, Spinal Fusion

## Abstract

**Background:**

Postoperative pain following lumbar spine surgery (LSS) can significantly impact recovery and patient satisfaction. Ultrasound-guided regional techniques such as quadratus lumborum plane block (QLPB) and caudal block (UGCB) have been proposed to enhance postoperative analgesia while reducing opioid consumption and opioid-related adverse effects (AEs).

**Objectives:**

To compare the analgesic efficacy and safety of ultrasound-guided QLPB versus ultrasound-guided caudal block in adult lumbar spine stabilization surgery.

**Methods:**

This randomized, double-blinded, prospective comparative study included 111 adult patients (18 - 60 years, ASA physical status I - II) scheduled for elective lumbar spine stabilization surgery. Patients were randomly allocated into three equal groups: Control (standard analgesia), ultrasound-guided caudal block (UGCB), or ultrasound-guided QLPB. Blocks were performed under ultrasound guidance after induction of general anesthesia using 0.25% bupivacaine. The primary outcome was the time to first postoperative rescue analgesia. Secondary outcomes included postoperative pain scores using the Numerical Pain Rating Scale (NPRS), total 24-hour postoperative opioid (meperidine) consumption, intraoperative opioid requirements, hemodynamic variables, and block- or opioid-related adverse events over the first 24 postoperative hours.

**Results:**

The time until the initial rescue analgesia was significantly delayed with QLPB in comparison to control and Caudal, with medians of 15, 1, and 5 h, respectively (P < 0.001). The total 24-hour (meperidine) intake was significantly diminished with QLPB as opposed to Caudal and control (P < 0.05). Numerical Pain Rating Scale scores were significantly diminished in QLPB and Caudal as opposed to control at 30 min, 1, 2, 4, 8, and 24 h (P < 0.001), with comparability detected at 12 h and 18 h. Intraoperative fentanyl administration was markedly diminished in the QLPB and Caudal groups, in contrast to the control group (P < 0.001). The occurrence of nausea and vomiting exhibited comparability across groups.

**Conclusions:**

Both ultrasound-guided caudal block and QLPB significantly improved postoperative analgesia compared with standard analgesic management following lumbar spine stabilization surgery. However, QLPB provided longer-lasting analgesia and was associated with lower postoperative opioid consumption than caudal block, highlighting its advantage for prolonged postoperative pain control.

## 1. Background

Postoperative pain following surgery is frequently aggravated by inadequate analgesic management, a problem of particular relevance in lumbar spine procedures (1). Effective postoperative analgesia after lumbar spine surgery (LSS) is associated with improved surgical outcomes, diminished hospital length of stay (LOS), and a diminished risk of progression to chronic pain (2). Nevertheless, opioid-based analgesia is linked to substantial adverse effects (AEs), including respiratory depression (RD), cognitive dysfunction, cardiovascular (CV) compromise, late wound healing, and gastrointestinal (GI) disturbances (3). Two regional techniques commonly employed for postoperative analgesia are the quadratus lumborum plane block (QLPB) and the ultrasound-guided caudal block (UGCB) (4). The UGCB targets the injection of local anesthetic (LAs) into the caudal epidural space at the sacral base in order to interrupt nociceptive transmission from the lower back and pelvis (5). Quadratus lumborum plane block aims to offer analgesia for lower abdominal and lumbar procedures via deposition of LAs in the fascial plane adjacent to the quadratus lumborum muscle (QLM), thereby targeting nerves that supply the abdominal wall (6, 7).

Although both techniques have demonstrated analgesic benefit in lumbar and lower abdominal surgery, no study has directly compared their relative efficacy in adult lumbar spine stabilization. Despite targeting different anatomical regions, QLPB through thoracolumbar fascial spread and UGCB through epidural distribution, both modalities may influence nociceptive transmission from lumbar and sacral segments relevant to spine surgery.

While both ultrasound-guided caudal block and QLPB are used for postoperative analgesia in truncal and lower abdominal surgery, their comparative efficacy in adult lumbar spine stabilization surgery, a procedure with distinct nociceptive drivers, remains unknown. Although UGCB provides analgesia through epidural spread to lumbosacral nerve roots and QLPB may offer broader somatic and visceral analgesia via thoracolumbar fascial and paravertebral spread, no direct comparison between these techniques has been conducted in this surgical population. Therefore, a head-to-head comparison is clinically warranted to evaluate their relative analgesic efficacy, opioid-sparing effects, and safety profiles following lumbar spine stabilization.

## 2. Objectives

The primary objective of this study was to compare the analgesic efficacy of ultrasound-guided QLPB and ultrasound-guided caudal block (UGCB) in adult patients undergoing lumbar spine stabilization surgery, as measured by the time to first postoperative rescue analgesia. The secondary objectives were to compare QLPB and UGCB with standard analgesic management regarding postoperative pain intensity [Numerical Pain Rating Scale (NPRS)], total postoperative opioid consumption during the first 24 hours, intraoperative opioid requirements, perioperative hemodynamic stability, and the incidence of block- or opioid-related adverse events, including postoperative nausea and vomiting.

## 3. Methods

This study adheres to the CONSORT reporting guidelines. This randomized, double-blind, controlled study was conducted at Fayoum University Hospital in accordance with the principles enumerated in the Declaration of Helsinki, following approval by the Fayoum University Hospital institutional ethics committee and local IRB (No. M696). All participants submitted written informed consent prior to enrollment and randomization. The study was registered on ClinicalTrials.gov (NCT06398600; in accordance with ICMJE standards).

The study comprised individuals aged 18 to 60 who were scheduled for elective lumbar spine fixation (LSF) and had a physical status of I or II, as defined by the American Society of Anesthesiologists (ASA). Refusal; contraindications to regional anesthesia (RA) (coagulopathy, injection site infection); previous lumbar disc surgery or spinal deformity; substance misuse; psychiatric disorders; allergy to LAs; and BMI > 35 kg/m² were the exclusion criteria.

Participants were randomly assigned to one of three groups (n = 37 each): Control (no block), caudal block (UGCB), or posterior QLPB, in a 1:1:1 ratio. A computer-generated sequence was used to randomize allocations, which were sealed in opaque envelopes and unsealed immediately prior to block performance. The investigation was administered on a patient- and assessor-blinded basis, as the performing anesthesiologist was unable to be blinded to the block technique. Through standardized draping and probe/needle placement, patients and outcome assessors were blinded to group assignment. To prevent unblinding, perioperative personnel were instructed to withhold block details, and independent anesthesiologists conducted postoperative assessments.

Noninvasive blood pressure, pulse oximetry, five-lead ECG, and capnography comprised the standard monitoring protocol. After five minutes of preoxygenation, propofol 2 mg/kg, fentanyl 1 µg/kg, and atracurium 0.5 mg/kg were administered to induce general anesthesia. Isoflurane (1.2 - 1.5%) was administered in air–oxygen to maintain anesthesia, and atracurium 0.1 mg/kg was administered every 20 minutes. In each instance, intravenous (IV) access was ensured.

Blocks were performed in the prone position following induction and hemodynamic stabilization via a LOGIQ P7 US system (GE Healthcare) with antiseptic covers and lubricant. Sensory block assessment was not feasible because all blocks were performed after induction of general anesthesia; therefore, dermatomal mapping could not be conducted.

The sacrococcygeal ligament and dorsal sacrum were identified by transversally positioning a linear 7 - 13 MHz transducer (or curvilinear 2 - 5 MHz in obesity) at the sacral hiatus, and subsequently rotating it longitudinally. Limiting advancement to ≤ 5 mm beyond the hiatus, an in-plane needle technique was implemented to penetrate the sacrococcygeal ligament and enter the caudal canal. The appropriate spread was confirmed by the unidirectional flow on color Doppler in the longitudinal view, following the administration of a 15 mL injection of 0.25% bupivacaine (AstraZeneca, Södertälje, Sweden) following negative aspiration (8, 9).

A posterior QLPB (QLB type 2) was performed under ultrasound guidance and a sterile curvilinear ultrasound probe was positioned transversely in the triangle of Petit, between the costal margin and the iliac crest, to perform a posterior QLPB. The probe was then advanced posteriorly to visualize the QLM and the middle thoracolumbar fascia adjacent to the posterior aponeurosis of the transversus abdominis muscle. Using an in-plane technique, a needle was advanced into the fascial plane between the QLM and the middle thoracolumbar fascia. Following hydrodissection with 2–3 mL of saline to confirm correct needle tip placement, 15 mL of 0.25% bupivacaine was injected on each side to achieve a bilateral block (10-13).

Intravenous fentanyl at a dose of 0.5 µg/kg was administered to patients with inadequate intraoperative analgesia, which was defined as a >20% elevation in heart rate (HR) or mean blood pressure (MAP) from baseline (cumulative dose recorded). Thirty minutes prior to skin closure, all patients were administered 1 g of IV paracetamol and 4 mg of IV ondansetron. IV neostigmine 0.05 mg/kg and atropine 0.01 mg/kg were administered to reverse neuromuscular blockade at the conclusion of the surgery. The patients were transferred to the PACU for the purpose of monitoring their MAP, HR, SpO₂, and respiratory rate (RR).

On admission to the PACU and at 1, 2, 4, 6, 8, 12, 18, and 24 hours postoperatively, the NPRS (0 - 10) was employed to evaluate analgesia outcomes. Intravenous meperidine 0.5 mg/kg was administered as rescue analgesia for NPRS ≥ 4 (meperidine was used in accordance with local institutional practice; however, we acknowledge that it is not part of many contemporary ERAS-based protocols). The time to first rescue and the cumulative 24-hour meperidine consumption were recorded. Over the course of 24 hours, hemodynamic parameters and intraoperative fentanyl supplementation were monitored every 30 minutes during the procedure and every 2 hours in the postoperative period. Systemic LA toxicity, hypotension, lethargy, paralysis, PONV, and neurological symptoms were prospectively documented. The duration of the operation was recorded.

The primary endpoint was the time to the initial rescue analgesia. Numerical Pain Rating Scale over 24 hours, total 24-hour meperidine consumption, additional intraoperative fentanyl, intra- and postoperative hemodynamics, complications, and operative duration were the secondary endpoints.

In order to identify a clinically significant difference in the primary outcome (time to first rescue), the sample size was determined via G*Power 3.1.9.6. The effect size was 0.68, as calculated by Ipek and co-authors (14) (mean difference 2.89 h; group SDs 1.94 and 5.71). In order to account for attrition, a minimum of 35 participants per group was necessary, with α = 0.05 (two-sided), power (1 − β) = 0.80, and equal allocation (1:1:1). A total of 37 participants per group were enrolled. The primary endpoint was the focus of the calculation in order to reduce Type II error. Secondary endpoints were regarded as exploratory, and multiplicity adjustments were implemented as needed.

IBM SPSS Statistics v22 (IBM Corp., Armonk, NY, USA) was employed to conduct statistical analyses. The Shapiro-Wilk and Kolmogorov-Smirnov tests were implemented to evaluate normality. In contrast to Kruskal-Wallis tests, continuous variables that are normally distributed are reported as mean ± SD and compared via a one-way ANOVA. Non-normal variables are reported as median (IQR). Categorical data were utilized in contrast to χ² tests. Statistical significance was indicated by a two-sided P-value of less than 0.05. Bonferroni correction was implemented for post-hoc between-group comparisons subsequent to a significant omnibus test.

The raw data supporting the findings of this study are available from the corresponding author upon reasonable request.

## 4. Results

**Figure 1.**
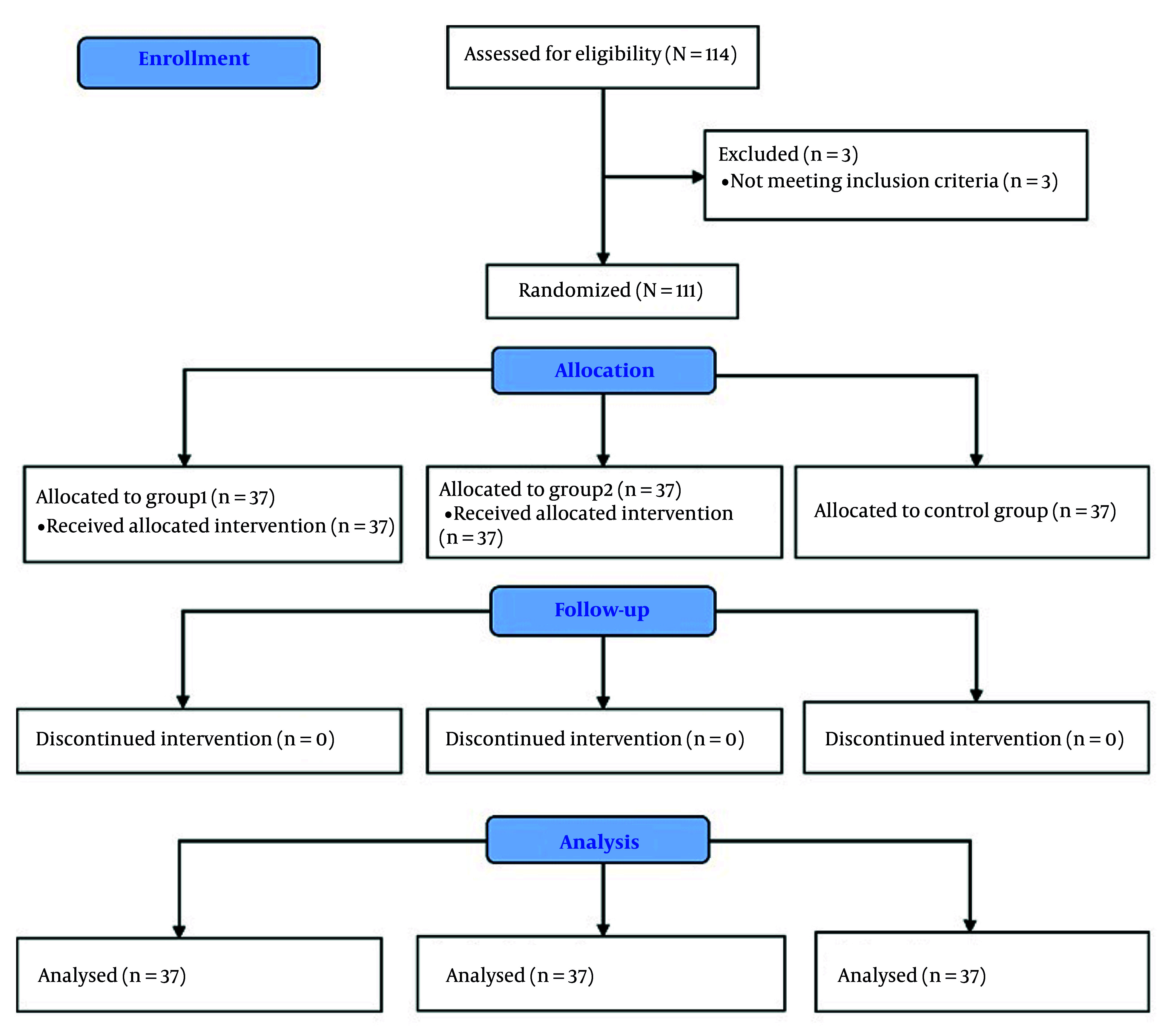
CONSORT flow diagram of patient enrollment, randomization, allocation, and analysis

One hundred eleven patients were equally randomized into three groups. Figure 1 illustrates the CONSORT flow diagram demonstrating patient enrollment, randomization, group allocation, and inclusion in the final analysis (Figure 1). Baseline demographic and surgical variables exhibited comparability among groups.

### 4.1. Analgesic Requirements

**Figure 2.**
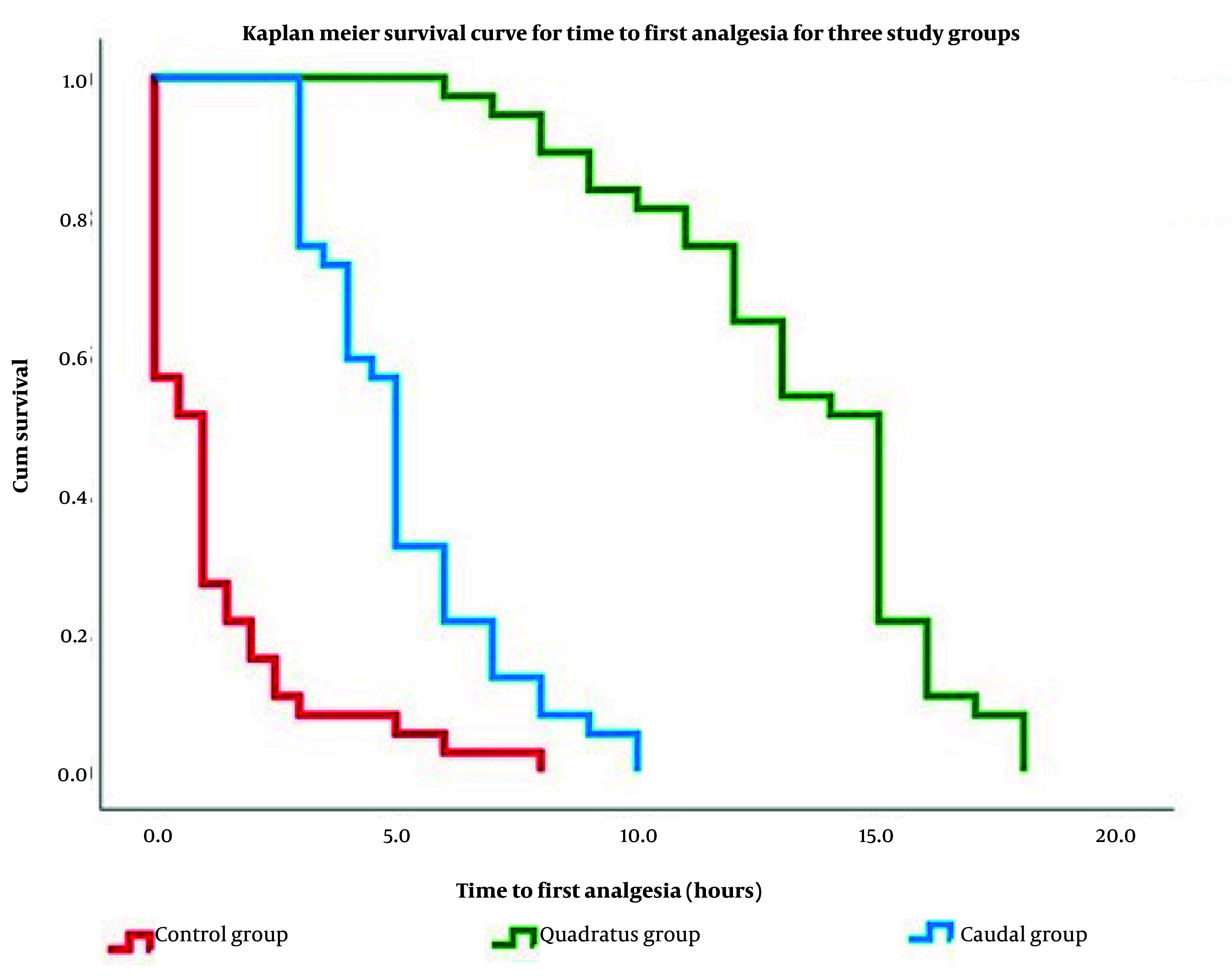
Time to first rescue analgesic (meperidine) among the investigated groups

Time to first rescue analgesia differed significantly between groups. Median time to first rescue was longest in the QLPB group (15.0 h) as opposed to the UGCB (5.0 h) and the control groups (1.0 h) (P < 0.001). The UGCB group also showed a significantly longer interval to first rescue as opposed to the control group. Figure 2 depicts the time to first rescue analgesia among the three study groups, highlighting the prolonged analgesic duration observed in the QLPB group compared with the caudal block and control groups. Total 24-h meperidine consumption was lowest in the QLPB group (median 50 mg), intermediate in the UGCB group (median 80 mg), and highest in the control group (median 150 mg) (P < 0.001). (Table 1 and Figure 2.) 

**Table 1.** Demographic Characteristics, Surgical Variables, and Analgesic Outcomes Among the Study Groups (N = 37) ^a, b^

Variables	Control Group	Quadratus Lumborum Group	Caudal Group	Difference Between Groups (95% CI)	P-Value ^c^
**Age (y)**	45.57 ± 9.29	43.62 ± 10.2	42.51 ± 11.1	M1 = 1.9 (-3.7 to 7.5); M2 = 3.05 (-2.6 to 8.7); M3 = 1.11 (-4.54 to 6.7)	0.432
**Gender**				RR = 1.06 (0.67 to 1.67)	0.897
Male	18 (48.65)	19 (51.35)	20 (54.05)		
Female	19 (51.35)	18 (48.65)	17 (45.95)		
**BMI (kg/m** ^**2**^ **)**	31.57 ± 1.32	31.17 ± 1.77	31.41 ± 1.89	M1 = 2 (0.56 to -0.5); M2 = 3 (0.9 to -0.7); M3 = 3 (0.82 to -1.1)	0.548
**ASA**				RR = 1.17 (0.86 to 1.58)	0.227
I	24 (64.86)	28 (75.68)	21 (56.76)		
II	13 (35.14)	9 (24.32)	16 (43.24)		
**Duration of surgery (min)**	192.2 ± 31.5	190.9 ± 28.5	194.3 ± 30.5	M1 = -1 (-15.4 to 17.9); M2 = -2 ( -18.8 to 14.5) M3 = -3 (-20 to 13.3)	0.888
**Time of first rescue Analgesic dose (h)**	1 ^A^ (0 - 1.5)	15 ^B^ (11.5 - 15)	5 ^C^ (3.25 - 6)	M1 = 13 (11.5 to 14); M2 = 4 (3 to 5); M3 = -9 (-10 to -7)	< 0.001 ^d^
**First analgesic pethidine (meperidine) dose (mg)**	40 ^A^ (30 - 50)	30 ^B^ (27.5 - 40)	30 ^C^ (30 - 40)	M1 = -10 (-10 to 0); M2 = -10 (-10 to -5); M3 = -0 (-5 to 0)	< 0.001 ^d^
**24 hours total postoperative pethidine (meperidine) consumption (mg)**	150 ^A ^(90 - 160)	50 ^B^ (40 - 60)	80 ^C^ (60 - 100)	M1 = -80 (-100 to -60); M2 = -45 (-70 to -30); M3 = 50 (40 to 55)	< 0.001 ^d^

Abbreviations: BMI, Body Mass Index; ASA, American Society of Anesthesiologists; IQR, interquartile range.

^a^ Values are expressed as mean ± SD for normally distributed variables and median (IQR) for non-normally distributed variables.

^b^ Different superscript capital letters (A, B, C) indicate statistically significant differences between groups according to post-hoc Bonferroni test.

^c^ Kruskal-Wallis test.

^d^ P < 0.05.

### 4.2. Pain Assessments

Numerical Pain Rating Scale scores were significantly diminished in both the QLPB and UGCB groups as opposed to control at PACU admission and at 30 min, 1, 2, 4, 8, and 24 h postoperatively (all P < 0.001). No significant intergroup differences in NPRS were detected at 12 and 18 h. (Table 2.) 

**Table 2.** Postoperative Numeric Pain Rating Scale Scores Over 24 Hours (N = 37) ^a^

Postoperative Timepoint	Control Group	Quadratus Lumborum Group	Caudal Group	Median Difference (95%CI)	P-Value ^b^
**After 30 minutes**	3 (2 - 5)	0 (0 - 1)	1 (0 - 2)	M1 = -2 (-4 - -2); M2 = -2 (-3 - -1); M3 = 1(0 - 1)	< 0.001 ^c^
**1 hour postoperative**	2 (2 - 3.5)	1 (0 - 1)	1 (1 - 2)	M1 = -2 (-2 - -1); M2 = -1 (-2 - -1); M3 = 1 (0 - 1)	< 0.001 ^c^
**P ^d^**	0.732	0.003 ^c^	0.025 ^c^		
**2 hours postoperative**	2 (2 - 3)	1 (1 - 2)	2 (1 - 2)	M1 = -1 (-2 - -1); M2 = -1 (-1 - 0); M3 = 1 (0 - 1)	< 0.001 ^c^
**P ^d^**	0.209	< 0.001^ c^	< 0.001 ^c^		
**4 hours postoperative**	3 (2 - 3)	2 (1 - 2)	2 (2 - 3)	M1 = -1 (-2 - -1); M2 = 0 (-1 - 0); M3 = 1 (0 - 1)	< 0.001 ^c^
**P ^d^**	0.577	< 0.001 ^c^	< 0.001 ^c^		
**8 hours postoperative**	3 (2.5 - 5.5)	2 (2 - 3)	2 (2 - 3)	M1 = -1 (-2 - -1); M2 = -1 (-2 - -1); M3 = 0 (0 - 1)	< 0.001 ^c^
**P ^d^**	0.223	< 0.001 ^c^	< 0.001 ^c^		
**12 hours postoperative**	3 (2 - 5)	3 (2 - 3)	3 (2 - 4.5)	M1 = 0 (-1 - -0); M2 = 0 (-1 - 1); M3 = 1( 0 - 1)	0.225
**P ^d^**	0.106	< 0.001 ^c^	< 0.001 ^c^		
**18 hours postoperative**	3 (2.5 - 5)	3 (2 - 4)	4 (2 - 5)	M1 = -1 (-1 - 0); M2 = 0 (-1 - 1); M3 = 0 (0 - 1)	0.124
**P ^d^**	0.317	< 0.001 ^c^	< 0.001 ^c^		
**24 hours postoperative**	5 (3 - 6)	2 (2 - 4)	3 (2 - 5)	M1 = -2 (-3 - 1); M2 = -1 (-2 - 0); M3 = 0 (0 - 1)	< 0.001 ^c^
**P ^d^**	0.077	< 0.001 ^c^	< 0.001 ^c^		

Abbreviation: IQR, interquartile range.

^a^ Values are expressed as median (IQR); IQR (25th-75th percentile).

^b^ Kruskal-Wallis test.

^c^ P < 0.05.

^d^ Post-hoc Bonferroni pairwise comparison between groups.

### 4.3. Intraoperative Opioid Use

The control group required supplemental intraoperative fentanyl (median 50 µg), whereas neither the QLPB nor the UGCB groups required additional intraoperative fentanyl (P < 0.001). (Table 3.) 

**Table 3.** Comparison of Supplemental Intraoperative Fentanyl Requirements Among Study Groups (N = 3) ^a^

Variable	Control Group	Quadratus Lumborum Group	Caudal Group	Median Difference (95% CI) ^a^	P-Value ^b^
**Additional Intraoperative fentanyl dose (microgram)**	50 (0 - 100)	0 (0 - 0)	0 (0 - 0)	M1 = -50 (-50 to -50); M2 = -50 (-50 to -50); M3 = 0 (0 to 0)	< 0.001 ^c^

Abbreviation: IQR, Interquartile range.

^a^ Values are expressed as median (IQR); IQR (25th-75th percentile).

^b^ Kruskal-Wallis test.

^c^ P < 0.05.

### 4.4. Complications and Adverse Events

The incidence of PONV was low and exhibited comparability among groups (P = 0.365). No cases of LA systemic toxicity, new neurological deficits, or other major AEs were recorded during the 24-h observation period. (Table 4.) 

**Table 4.** Incidence of Postoperative Complications Among Study Groups (N = 3) ^a^

Variables	Control Group	Quadratus Lumborum Group	Caudal Group	P-Value ^b^
**Nausea**	0 (0)	1 (3)	0 (0)	0.365
**Vomiting**	0 (0)	1 (3)	0 (0)	0.365

^a^ Values are expressed as No. (%).

^b^ Chi-square test.

### 4.5. Hemodynamics

**Figure 3.**
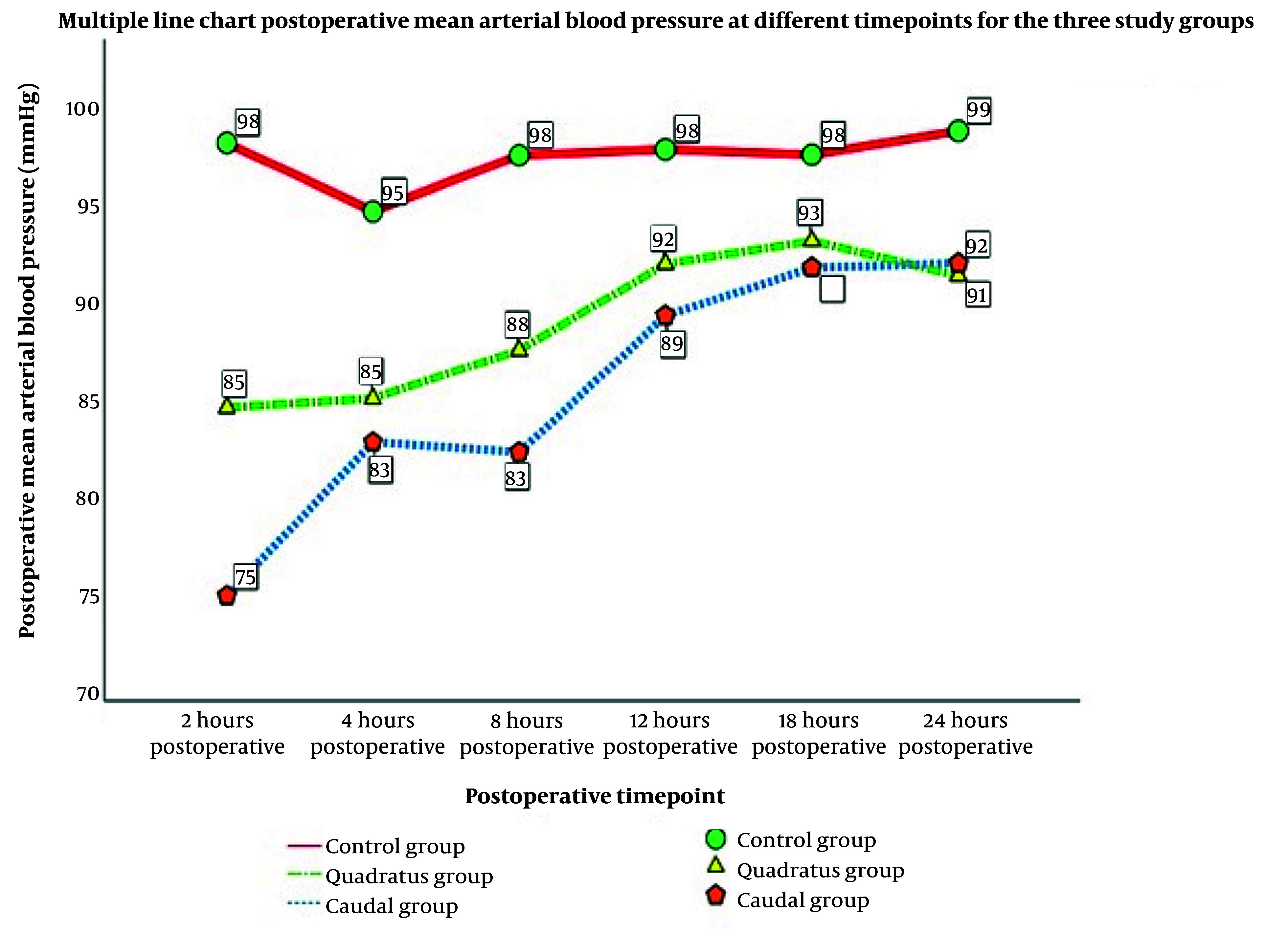
A, intraoperative mean arterial blood pressure trends among the investigated group; B, postoperative mean arterial blood pressure trends during the first 24 hours (Abbreviation: MAP, mean arterial pressure)

HR and MAP remained stable and comparable across groups during the intraoperative period and throughout postoperative monitoring; Figure 3 shows intraoperative and postoperative mean arterial pressure trends over the first 24 hours, demonstrating comparable hemodynamic stability across the three groups (Figure 3). 

## 5. Discussion

Regional anesthesia techniques have been increasingly incorporated into multimodal analgesic strategies for LSS to improve postoperative pain control and reduce opioid consumption (15, 16). In the present randomized controlled study, both ultrasound-guided caudal block and QLPB significantly improved postoperative analgesia compared with standard management. However, QLPB was associated with a longer duration of postoperative analgesia and reduced opioid requirements, suggesting a potential advantage for sustained pain control following lumbar spine stabilization.

Caudal block provides analgesia primarily through epidural spread to the lumbosacral nerve roots and has been shown to reduce perioperative opioid requirements in selected lumbar procedures (17). Previous studies have demonstrated the analgesic efficacy of ultrasound-guided regional techniques in spine and lower abdominal surgery (5, 18-20). Although ultrasound guidance improves the accuracy and safety of caudal block performance (21, 22), its effectiveness in adult patients remains variable, and concerns regarding neurological complications persist (23, 24). The QLPB, a fascial plane block of the posterior abdominal wall, has shown efficacy in managing postoperative pain, affirming its value for postoperative analgesia (25, 26).

Numerous studies have thoroughly shown the significance of efficient postoperative pain management in improving patient outcomes, especially with early mobility and less narcotic use. For example, in single-level lumbar decompression operations, Saoud and co-authors (15) showed that pre-emptive caudal bupivacaine-morphine significantly prolonged the duration of postoperative analgesia, therefore diminishing the need for intra- and postoperative analgesics. This technique was effective in expediting recovery and diminishing reliance on additional analgesics by facilitating quicker patient ambulation without inducing significant hemodynamic instability or an elevation in AEs.

Similarly, SERTCAKACILAR and co-authors (16) indicated that patients receiving QLPB for postoperative analgesia had diminished opioid use and pain intensity during the first 24 h following surgical intervention. The QLPB was linked to enhanced early mobility and decreased hospital durations, both essential for cost savings and expedited recovery. Nevertheless, they noted that opioid use associated with the QLPB escalated by the 24th h, presumably owing to the declining efficacy of the long-acting LAs administered. These results underscore the need to choose suitable analgesic treatments that offer prolonged analgesia to optimize patient outcomes in the postoperative phase.

The extended analgesia after a single-shot QLPB may indicate significant cranio-caudal and anterior diffusion into the paravertebral/thoracolumbar area, resulting in a more comprehensive somatic and visceral block (9). The thoracolumbar fascia serves as a conduit and a slow-release reservoir for injectate, therefore delaying absorption and prolonging LA exposure (27, 28).

This study has several limitations. First, it was conducted at a single center, which may limit generalizability because surgical and anesthetic practices vary across institutions. Second, all regional blocks were performed after induction of general anesthesia, which precluded sensory assessment and confirmation of block success. This represents a major limitation of the study, as block failure rates, particularly for ultrasound-guided caudal block in adult patients, could not be determined. Consequently, variability in block success may have contributed to the observed differences in analgesic outcomes, and some effects may reflect differences in block effectiveness rather than true comparative efficacy. In addition, the QLPB was performed bilaterally and therefore involved a higher total volume and dose of LAs compared with the caudal block. This difference in total LAs exposure may have contributed to the longer duration of analgesia and reduced opioid consumption observed in the QLPB group and represents a potential confounding factor. Future studies should consider performing blocks before induction of general anesthesia or incorporating objective methods to confirm block success. Third, the follow-up period was limited to the first 24 postoperative hours. Consequently, important outcomes such as rebound pain after block resolution, longer-term recovery trajectory, and the development of persistent or chronic postoperative pain could not be evaluated. Fourth, pain assessments were performed only at rest, which may underestimate the clinical impact of each block during movement. Fifth, Pethidine (meperidine) was used as the primary rescue opioid. Although Pethidine (meperidine) was the only opioid analgesic routinely available at our institution during the study period, it is not included in most contemporary multimodal or ERAS analgesic protocols because of its unfavorable safety profile and the neurotoxic potential of its metabolite (normeperidine). This may limit the external validity and generalizability of our findings to centers using alternative opioids such as morphine, hydromorphone, or fentanyl; however, its consistent use across all study groups preserves internal comparative validity. Future studies should include dynamic pain measures, extended follow-up, and multicenter designs to improve generalizability and better characterize the analgesic profiles of these techniques.

Despite these limitations, the study has notable strengths. The randomized, double-blind, controlled design enhances methodological rigor and reduces bias. The sample size was determined through a priori power analysis, and participant retention was complete. This trial provides one of the earliest direct comparisons between QLPB and UGCB in adult lumbar spine stabilization, addressing a meaningful gap in the literature. Objective outcomes including time to first rescue analgesia, opioid consumption, intraoperative fentanyl use, and hemodynamic trends support the robustness of the findings. These strengths offer valuable evidence to support the integration of regional anesthesia into multimodal analgesic strategies for LSS.

In conclusion, ultrasound-guided regional anesthesia techniques can effectively and safely enhance postoperative analgesia following lumbar spine stabilization surgery. Both ultrasound-guided caudal block and QLPB reduced perioperative opioid requirements and improved pain control compared with standard analgesic management. Notably, QLPB was associated with a significantly longer duration of postoperative analgesia and lower postoperative opioid consumption than caudal block. These findings support the integration of regional anesthesia into multimodal analgesic strategies for LSS, with QLPB offering particular advantages for sustained postoperative analgesia.

## Data Availability

The dataset presented in the study is available on request from the corresponding author during submission or after publication.

## Clinical Trial Registration Code:

NCT06398600.
